# [^18^F]FE@SUPPY: a suitable PET tracer for the adenosine A3 receptor? An in vivo study in rodents

**DOI:** 10.1007/s00259-014-2976-3

**Published:** 2015-01-20

**Authors:** Daniela Haeusler, Claudia Kuntner, Lukas Nics, Markus Savli, Markus Zeilinger, Thomas Wanek, Panagiotis Karagiannis, Rupert R. Lanzenberger, Oliver Langer, Karem Shanab, Helmut Spreitzer, Wolfgang Wadsak, Marcus Hacker, Markus Mitterhauser

**Affiliations:** 1 Department of Nuclear Medicine, Medical University of Vienna, 1090 Vienna, Austria; 2 Biomedical Systems, Health & Environment Department, AIT Austrian Institute of Technology GmbH, 2444 Seibersdorf, Austria; 3 Department of Nutritional Sciences, University of Vienna, 1090 Vienna, Austria; 4 Department of Psychiatry and Psychotherapy, Medical University of Vienna, 1090 Vienna, Austria; 5 Cutaneous Medicine and Immunotherapy, St. John’s Institute of Dermatology, Division of Genetics and Molecular Medicine King’s College London School of Medicine, Guy’s Hospital, King’s College London, London, UK; 6 Department of Drug and Natural Product Synthesis, University of Vienna, 1090 Vienna, Austria

**Keywords:** Adenosine A3 receptor, Small-animal PET, [^18^F]FE@SUPPY, Xenograft, Efflux transporter, Tumour marker

## Abstract

**Purpose:**

The adenosine A_3_ receptor (A3R) is involved in cardiovascular, neurological and tumour-related pathologies and serves as an exceptional pharmaceutical target in the clinical setting. A3R antagonists are considered antiinflammatory, antiallergic and anticancer agents, and to have potential for the treatment of asthma, COPD, glaucoma and stroke. Hence, an appropriate A3R PET tracer would be highly beneficial for the diagnosis and therapy monitoring of these diseases. Therefore, in this preclinical in vivo study we evaluated the potential as a PET tracer of the A3R antagonist [^18^F]FE@SUPPY.

**Methods:**

Rats were injected with [^18^F]FE@SUPPY for baseline scans and blocking scans (A3R with MRS1523 or FE@SUPPY, P-gp with tariquidar; three animals each). Additionally, metabolism was studied in plasma and brain. In a preliminary experiment in a mouse xenograft model (mice injected with cells expressing the human A3R; three animals), the animals received [^18^F]FE@SUPPY and [^18^F]FDG. Dynamic PET imaging was performed (60 min in rats, 90 min in xenografted mice). In vitro stability of [^18^F]FE@SUPPY in human and rat plasma was also evaluated.

**Results:**

[^18^F]FE@SUPPY showed high uptake in fat-rich regions and low uptake in the brain. Pretreatment with MRS1523 led to a decrease in [^18^F]FE@SUPPY uptake (*p* = 0.03), and pretreatment with the P-gp inhibitor tariquidar led to a 1.24-fold increase in [^18^F]FE@SUPPY uptake (*p* = 0.09) in rat brain. There was no significant difference in metabolites in plasma and brain in the treatment groups. However, plasma concentrations of [^18^F]FE@SUPPY were reduced to levels similar to those in rat brain after blocking. In contrast to [^18^F]FDG uptake (*p* = 0.12), the xenograft model showed significantly increased uptake of [^18^F]FE@SUPPY in the tissue masses from CHO cells expressing the human A3R (*p* = 0.03). [^18^F]FE@SUPPY was stable in human plasma.

**Conclusion:**

Selective and significant tracer uptake of [^18^F]FE@SUPPY was found in xenografted mice injected with cells expressing human A3R. This finding supports the strategy of evaluating [^18^F]FE@SUPPY in “humanized animal models”. In conclusion, preclinical evaluation points to the suitability of [^18^F]FE@SUPPY as an A3R PET tracer in humans.

## Introduction

Adenosine is one of the main “speed limiters” in the human body contributing to most energy-associated processes. It unfolds its various effects via A_1_, A_2A_, A_2B_ and A_3_ receptors. Due to the late discovery of the adenosine A_3_ receptor (A3R) in the 1990s, quantitative data regarding its distribution and density in vivo is still sparse. Besides its broad involvement in cardiovascular and neurological pathologies, recent studies indicate a prevalent association of A3R in tumour-related diseases. A3R has been reported to be especially pronounced in prostate, breast and colon carcinoma and melanoma [1]. Surprisingly, although the involvement of A3R in tumour genesis, and cardiovascular and neurological pathologies is undisputed, no antagonists have so far reached clinical trials. Until recently, there were few radioligands for A3R available ([^125^I]AB-MECA, an A3 agonist, being one), and no PET ligands for the imaging and evaluation of A3R. Nevertheless, future clinical applications of suitable PET tracers can be envisaged, e.g. as tumour markers for colon, breast and prostate carcinoma and melanoma, which today are diseases with high mortality.

We therefore introduced the A3R antagonist [^18^F]FE@SUPPY (5-(2-[^18^F]fluoroethyl)-2,4-diethyl-3-(ethylsulfanylcarbonyl)-6-phenylpyridine-5-carboxylate) as the first PET tracer for A3R [2, 3]. Meanwhile, a second potential PET radiotracer has been presented, radiolabelled with ^76^Br [4]. Furthermore, we converted the fluoroethyl ester [^18^F]FE@SUPPY into the fluoroethyl thioester [^18^F]FE@SUPPY:2 (5-ethyl 2,4-diethyl-3-((2-[^18^F]fluoroethyl)-sulfanylcarbonyl)-6-phenylpyridine-5-carboxylate), to serve as a second potential A3R PET tracer [5, 6]. Recently, a series of ^11^C-labelled 1,2,4-triazolo[4,3-α]quinoxalin-1-one derivatives were synthesized as candidate PET radioligands for A3R [7].

[^18^F]FE@SUPPY displays high affinity for human A3R (*K*_i_ 4.2 nM), whereas its affinity towards rat A3R is rather low (*K*_i_ 600 nM) [8]. This fact can be explained by the species differences regarding the A3R protein, the difference between humans and rats being pronounced with a sequence homology of the proteins of only 72 % [9]. Furthermore, binding studies have revealed affinities of FE@SUPPY for human A1R and A2AR in the micromolar range, severalfold lower than towards human A3R [10].

Therefore the results of the preclinical evaluation of [^18^F]FE@SUPPY seemed quite promising: successful radiopreparation of [^18^F]FE@SUPPY with satisfactory yields and good specific radioactivity in an automated setup allowed a series of preclinical evaluations [2, 3]. The ex vivo biodistribution follows mainly the mRNA pattern of A3 described previously [3], except for the lack of pronounced tracer uptake in rat testes [11]. Ex vivo metabolite studies in rats have shown metabolites to a certain extent in the blood [12], and logP assessment has shown high lipophilicity of the title compound [6]. Autoradiographic in vitro competition experiments on human and rat brain tissues with [^125^I]AB-MECA have confirmed the comparability of the title compound FE@SUPPY and the commercially available and highly selective A3 antagonist MRS1523 [13]. Furthermore, biodistribution in rats shows that brain-to-blood ratios increase over time [3]. Hence, we aimed to investigate the in vivo behaviour of [^18^F]FE@SUPPY using small-animal PET in rats and xenografted mice. Additionally, keeping in mind the differences between these species, we wanted to assess the in vitro stability of [^18^F]FE@SUPPY in human and rat plasma. In detail, the aims of the study were:(1) baseline small-animal PET scans, (2) small-animal PET scans with A3R blocking, (3) examination of potential influence of the efflux system (P-gp), (4) investigations regarding a xenograft mouse model (with inoculated cells expressing the human A3R), and (5) in vitro stability of [^18^F]FE@SUPPY in human and rat plasma.

## Materials and methods

### Tracer preparation

Radiosynthesis of [^18^F]FE@SUPPY was performed in a fully automated synthesizer (TRACERlab Fx_FN_; GE Healthcare) as described elsewhere [2, 3].

### Chemicals

All chemicals were of analytical grade and obtained from Sigma-Aldrich Chemie GmbH (Schnelldorf, Germany) or Merck KGaA (Darmstadt, Germany). Isoflurane was obtained from Baxter Vertriebs GmbH (Vienna, Austria). Tariquidar dimesylate (TQD) was kindly provided by Dr. Erker (Department of Medicinal Chemistry, University of Vienna, Austria). Primary antibody (Anti-Adora3, HPA 028568) was obtained from Santa Cruz Biotechnology, CA; and the avidin-biotin-complex (Vectastain ABC kit PK 4001), aqueous mounting medium (VectaMount™) and AEC solution (AEC substrate kit for peroxidase, SK-4200) were purchased from Vector Laboratories, CA.

### Cell lines

The Chinese hamster ovary cell line CHO-K1 (CCL-61) was obtained from ATCC and the CHO cell line, stably expressing human A3R (CHO-A3) was a generous gift from Prof. Karl-Norbert Klotz (University of Würzburg, Germany) [14]. All cell lines were grown and handled according to standard protocols.

### Animals

The study was approved by the local Animal Welfare Committee and all study procedures were performed in accordance with the Austrian Animal Experiments Act (LF1-TVG-34/040-2008). All efforts were made to minimize pain and discomfort as well as the number of animals.

Adult male Sprague-Dawley rats (Him:OFA; Animal Research Institute, Himberg, Austria) weighing 307 ± 35 g were kept under controlled environmental conditions (22 ± 1 °C, 40 – 70 % humidity, 12-h light/dark cycle) with free access to standard laboratory animal diet and tap water. Before being used in the experiments, the animals were allowed to adapt to the new conditions for at least a week. Prior to each experiment, the animals were placed in a chamber containing 2.5 % isoflurane in oxygen. When unconscious, the animals were taken from the chamber and kept under anaesthesia with 1.8 % isoflurane administered via a mask during the whole experiment. Pathogen-free, female NMRI-nu/nu mice (Taconic, Denmark) weighing 28.1 ± 0.8 g were housed under sterile conditions. Mice were injected subcutaneously into both upper flanks with 100 μL of a cell suspension in phosphate-buffered saline containing 10^6^ CHO cells (right side) or 10^6^ CHO-A3 cells (left side). During the imaging periods of 60 or 90 min (rats or xenografted mice, respectively) the isoflurane level was adjusted depending on the depth of anaesthesia. A humidifier was used to moisten the gas mixture before supplying it to the animal.

### Small-animal PET

#### Experimental procedure

A microPET Focus220 scanner (Siemens, Medical Solutions) was used for the experiments. It has 168 detector modules providing a 7.6-cm axial and 22-cm transaxial field of view. The resolution of the reconstructed images (filtered back projection) was 1.3 mm (full-width at half-maximum) in the central field of view and remained under 2 mm within the central 5 cm diameter field of view [15]. For PET imaging two groups of animals (rats and xenografted mice) were investigated. Rats were divided into four groups. The first group received vehicle (dimethyl sulphoxide, DMSO) intravenously via a lateral tail vein 30 min prior to radiotracer administration (three animals, 14.3 – 70.1 MBq, 0.2604 ± 0.06 μg). The second group received MRS1523 – a selective A3R antagonist – at a dose of 2 mg/kg body weight (dissolved in DMSO, 4 mg/mL) intravenously via a tail vein 30 min prior to radiotracer administration (three animals, 18.8 – 59.1 MBq, 0.3443 ± 0.05 μg). The third group received unlabelled title compound FE@SUPPY (dissolved in DMSO, 4 mg/mL) at a dose of 2 mg/kg 30 min before tracer administration (three animals, 9.7 – 16.1 MBq, 0.3926 ± 0.04 μg). In the final study group, the effect of P-glycoprotein inhibition was assessed by intravenous administration of 15 mg/kg TQD (freshly dissolved on each experimental day in 2.5 % aqueous dextrose solution and injected intravenously via a tail vein at a volume of 3 mL/kg over 1 min) 60 min prior to tracer administration (three animals, 13.9 – 26.8 MBq, 0.7451 ± 0.11 μg).

Anaesthetized animals were positioned in the imaging chamber of the microPET scanner, which was kept at 38 °C throughout the experiment. A stereotactic holder attached to the bed, consisting of ear plugs and a tooth bar, was used to fix the animal's head to ensure a reproducible position during the PET studies. [^18^F]FE@SUPPY (27.91 ± 13.82 MBq) dissolved in 0.48 ± 0.22 mL phosphate-buffered saline (pH 7.4) was administered as a bolus via a tail vein. Dynamic PET imaging was performed over 60 min in all animals. At the end of the PET scan the animals were killed, a terminal blood sample was collected and whole brains were removed for testing of metabolite stability.

#### Xenograft mouse model

Around 17 days after cell inoculation, animals in the xenograft group were positioned in the imaging chamber and a dynamic 90-min PET scan was initiated at the start of [^18^F]FE@SUPPY injection (2.21 ± 0.75 MBq dissolved in 100 μl physiological saline, pH 7.4; three animals) via a tail vein under isoflurane anaesthesia (2 %). Additionally, in this group, [^18^F]FDG PET was performed 1 day prior to the [^18^F]FE@SUPPY experiments. As animal handling has been found to have a considerable impact on [^18^F]FDG PET results, it was standardized for all animals [16, 17]. In brief, mice were deprived of food for 6 – 8 h before [^18^F]FDG injection via a tail vein. Mice had access to drinking water at all times. Body warming was achieved by placing the entire cage on a heating pad kept at 38 °C. Warming was started around 30 min before tracer injection and was continued throughout the uptake and imaging period. [^18^F]FDG (6.03 ± 1.31 MBq dissolved in 100 μL physiological saline, pH 7.4; three animals) was injected via a tail vein under isoflurane anaesthesia (2 %). Mice were kept under isoflurane anaesthesia throughout the whole uptake and imaging period. A 10-min static image was acquired at 1 h after [^18^F]FDG injection. Dynamic PET imaging was performed over 90 min in xenografted animals. At the end of the PET scan the animals were killed and tumours were resected for staining purposes.

#### PET data analysis

For all PET scans (rats and xenografted mice), list mode data were acquired with an energy window of 350 – 750 keV (rats) or 250 – 750 keV (mice) and a 6-ns timing window. Before each PET scan, a transmission scan using a ^57^Co point source was recorded over 10 min. The PET data from the 60-min dynamic scans were sorted into three-dimensional sinograms according to the following frame sequence: 6 × 20 s, 5 × 60 s, 4 × 120 s, 3 × 300 s and 3 × 600 s. For the 90-min scans (in the xenografted mice) the following frame sequence was used: 8 × 5 s, 2 × 10 s, 2 × 30 s, 3 × 60 s, 2 × 150 s, 2 × 300 s, 4 × 600 s and 2 × 900 s. PET images were reconstructed by Fourier rebinning followed by filtered back projection with a ramp filter. The resulting voxel size was 0.6 × 0.6 × 0.8 mm for rats or 0.4 × 0.4 × 0.8 mm for mice. The standard data correction protocol (normalization, attenuation and decay correction) was applied to the data. A calibration factor for converting units of microPET images into absolute radioactivity concentration units was first generated by imaging a phantom filled with a known concentration of [^18^F]FDG.

Rat brains were manually outlined on multiple planes of the PET summation images using the image analysis software PMOD 3.1 (Pmod Ltd, Zürich, Switzerland) or Amide [18]. Volumes of interest in the brain or solid tissue mass (xenografts) were transferred to the PET images of the individual time frames and time–activity curves (TACs) expressed in units of kilobecquerels per millilitre were calculated. To facilitate comparison of TACs of different animals, radioactivity concentrations were normalized to dose and weight and expressed as standardized uptake value (SUV, %ID/cm^3^ or %ID/g). For each TAC, the area under the curve (AUC, %ID/cm^3^·min or %ID/g·min) from time zero to the last observation point was calculated using the OriginPro 7.5G software package (OriginLab Corporation, Northampton, MA).

### Metabolism

#### Ex vivo stability

To determine the influence of the four treatments (vehicle, MRS1523, FE@SUPPY, TQD) on uptake of [^18^F]FE@SUPPY, stability was assessed in plasma and brain 60 min after tracer injection. Final blood samples (3 mL) were collected in heparin-buffered tubes, rat brains were washed with ice-cold water and homogenized in 0.9 % aqueous NaCl solution (1 mL) using an IKA T10 basic Ultra-turrax (IKA Laboratory Equipment, Staufen, Germany). Blood and brain homogenate probes were each spiked with unlabelled FE@SUPPY (0.5 mg/ml), and centrifuged (4,000 rpm, 5 min) to separate cellular components. Sample clean-up was performed by vortexing plasma with the equivalent amount of methanol/acetonitrile (9:1) for 1 min and by subsequent ultracentrifugation (10,000 rpm, 5 min) to remove precipitated proteins. The supernatants obtained were analysed by radio-HPLC (Agilent, Boeblingen, Germany) equipped with a series 1100 autosampler, a series 1200 quaternary pump, a series 1200 diode array detector and a lead-shielded BGO radiodetector. A Chromolith® Performance RP-18e (100 × 4.6 mm, 5 μm) column with a Chromolith® Performance RP-18e (4 x4 mm, 5 μm) precolumn was eluted at a flow rate of 2 mL/min using a mobile phase consisting of 60 % acetonitrile and 40 % water/acetic acid 97.5:2.5 and 32 mM ammonium acetate, pH 3.5. UV detection was performed at a wavelength of 254 nm. The retention time of [^18^F]FE@SUPPY was 4.6 – 5.9 min (UV detection and radiodetection).

#### In vitro plasma stability

Experiments were conducted following a standard protocol [19]. Briefly, [^18^F]FE@SUPPY was added to lithium-heparinized human or rat plasma and after predetermined times (0, 60 and 120 min), the incubation mixture was eluted through a preconditioned SPE cartridge. The washed and newly eluted eluate solutions were injected into the radio-HPLC system (HPLC system as described in the section Ex vivo stability). The stationary phase was an analytical HPLC column LiChrospher LICHROCART® RP-18e (5 μm), 125 × 4 mm precolumn, and the mobile phase consisted of sodium citrate tribasic dehydrate (buffered at 0.034 M, pH 2.5)/acetonitrile 32:68 at a flow rate of 2 mL/min. Samples were incubated with a Thermomixer Compact from Eppendorf (Vienna, Austria) and centrifuged with a Universal 30 RF centrifuge from Hettich (Tuttlingen, Germany).

### Statistical analysis

All values are given as arithmetic means ± standard deviation (unless stated otherwise). To determine potential significant differences, a two-tailed *t* test with α = 0.95 and analysis of variance testing were performed using the statistics add-on in Windows® Excel 2010. A value of *P* ≤ 0.05 was considered significant.

## Results

### Tracer preparation

Starting from 51 ± 25G Bq of [^18^F]fluoride, 8 ± 2 GBq of formulated [^18^F]FE@SUPPY was achieved. Radiochemical purity was greater than 98 % and the specific activity at the end of synthesis was 61 ± 12 GBq/μmol. The only radioactive contaminant was [^18^F]fluoride. The identity of [^18^F]FE@SUPPY was confirmed by HPLC with coinjection with unlabelled reference compound and by thin-layer chromatography.

### Small-animal PET

#### Baseline

[^18^F]FE@SUPPY showed generally high uptake in fat-rich regions such as the harderian and lacrimal glands (consistent uptake over 60 min) and low uptake in the brain which decreased after 10 – 15 min.

#### Blocking

**Fig. 1:**
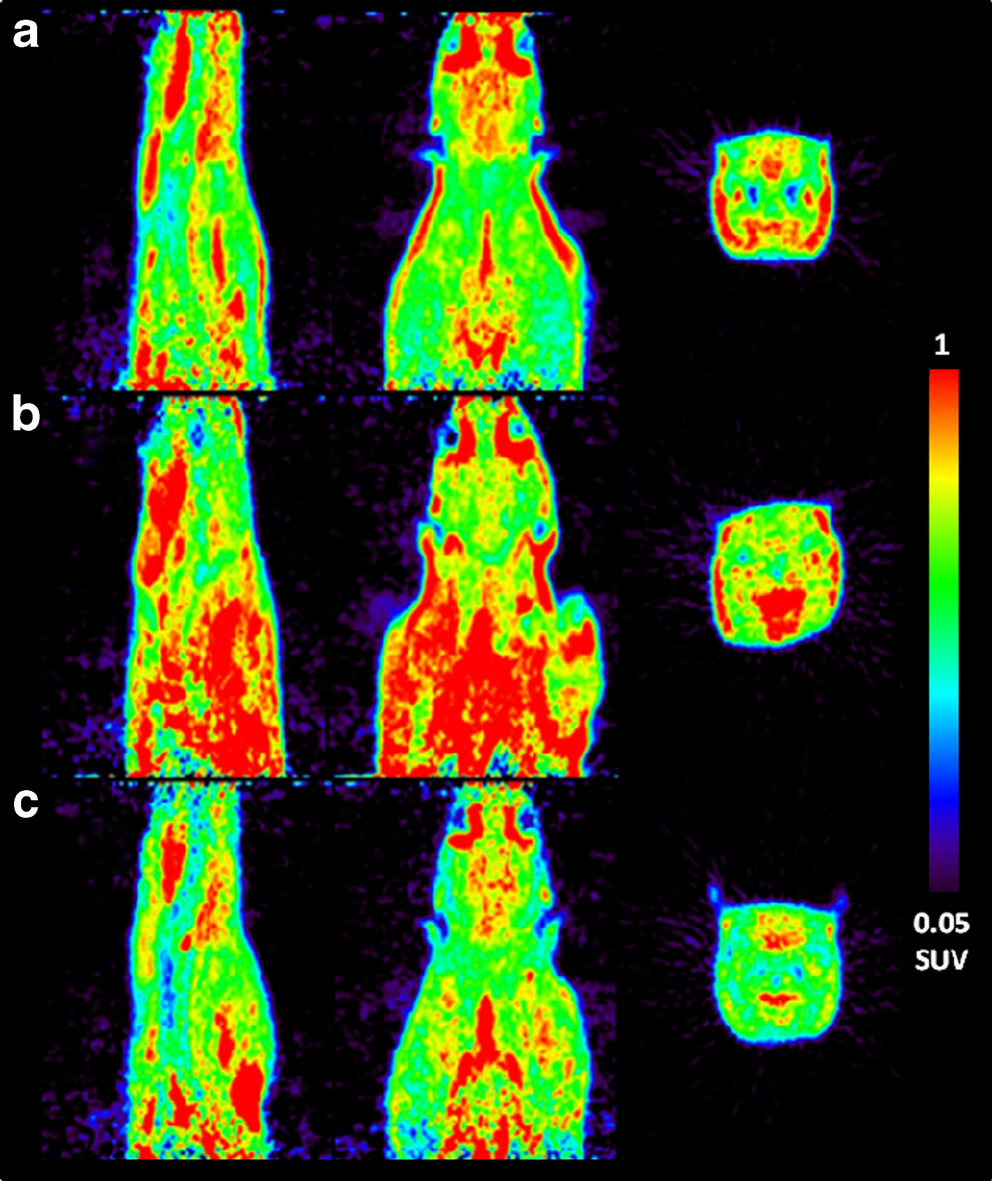
Sagittal, coronal and transverse images (summed over 60 min) showing uptake of [^18^F]FE@SUPPY in rat brain: **a** baseline scan with DMSO vehicle, **b** blocking with MRS1523 (2 mg/ml), **c** efflux inhibition with tariquidar (15 mg/ml)

**Fig. 2:**
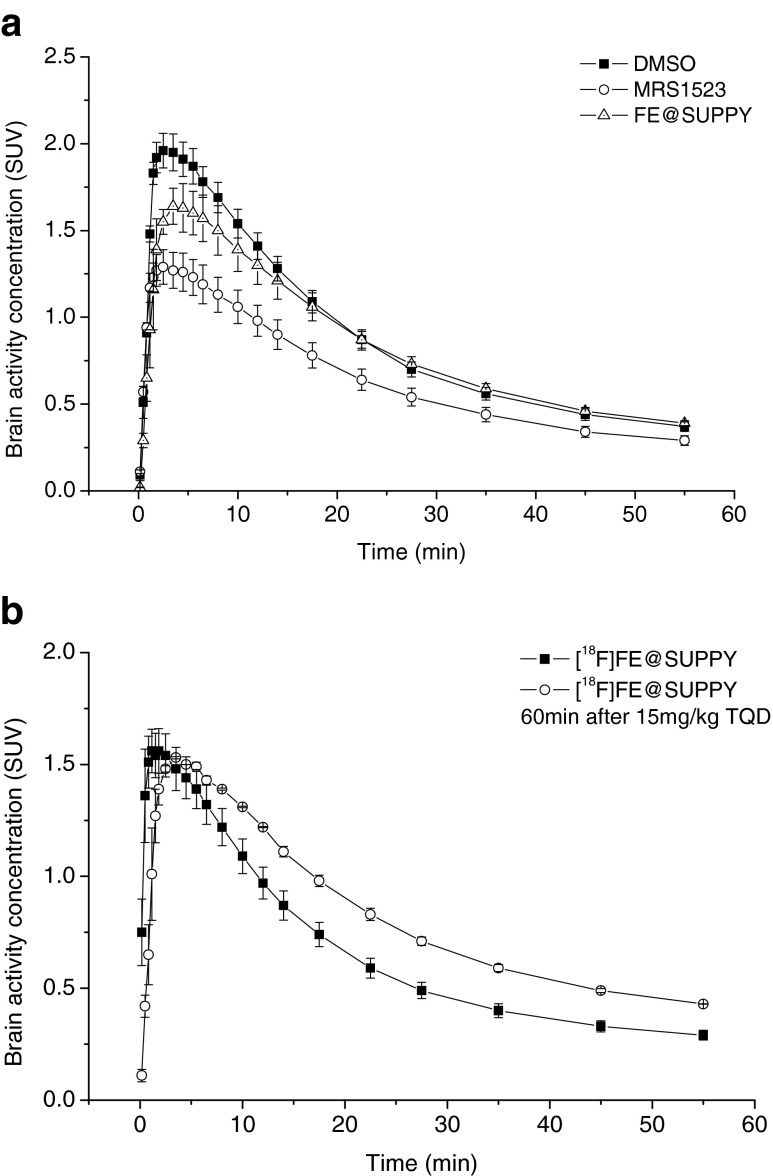
Time–activity curves (mean ± SEM) of [^18^F]FE@SUPPY uptake in rat brain (**a**) 30 min after adminstration of DMSO vehicle (*closed squares*), 2 mg/kg of MRS1523 (*open circles*) or 2 mg/kg of FE@SUPPY (*open triangles*; three animals per group) and (**b**) 60 min after adminstration of DMSO vehicle (*closed squares*) or 15 mg/kg of tariquidar (*open circles*; three animals per group)

Typical blocking scans of [^18^F]FE@SUPPY in male rats after pretreatment with DMSO (vehicle) and MRS1523 are shown in Fig. 1a, b. MRS1523 administration led to a significant reduction (*p* = 0.009) in peak brain activity of 34.1 % as compared with DMSO administration (Fig. 2a). Unlabelled FE@SUPPY administration led to a reduction in peak brain activity of 16.7 % as compared with DMSO administration, but the reduction did not reach statistical significance (*p* = 0.1, Fig. 2a). The AUCs followed the trend observed in the peak brain activities following all four pretreatments. A 1.35-fold higher AUC was found after DMSO administration as compared to no treatment (*p* = 0.016). MRS1523 administration led to a 27.6 % reduction in AUC (*p* = 0.03) as compared with DMSO administration. Unlabelled FE@SUPPY administration led to a 5.4 % reduction in AUCs as compared with DMSO administration.

#### Efflux inhibition

A typical blocking scan of [^18^F]FE@SUPPY in male rats after pretreatment with TQD is shown in Fig. 1c. Peak brain activity was similar in the untreated (1.56 ± 0.22 SUV) and the TQD-treated group (1.53 ± 0.01 SUV). Figure 2b shows brain TACs from untreated and TQD-treated rats. P-gp inhibition resulted in a 1.24-fold increase in the brain AUC relative to the baseline scan (*p* = 0.09).

#### Xenograft mouse model

**Fig. 3:**
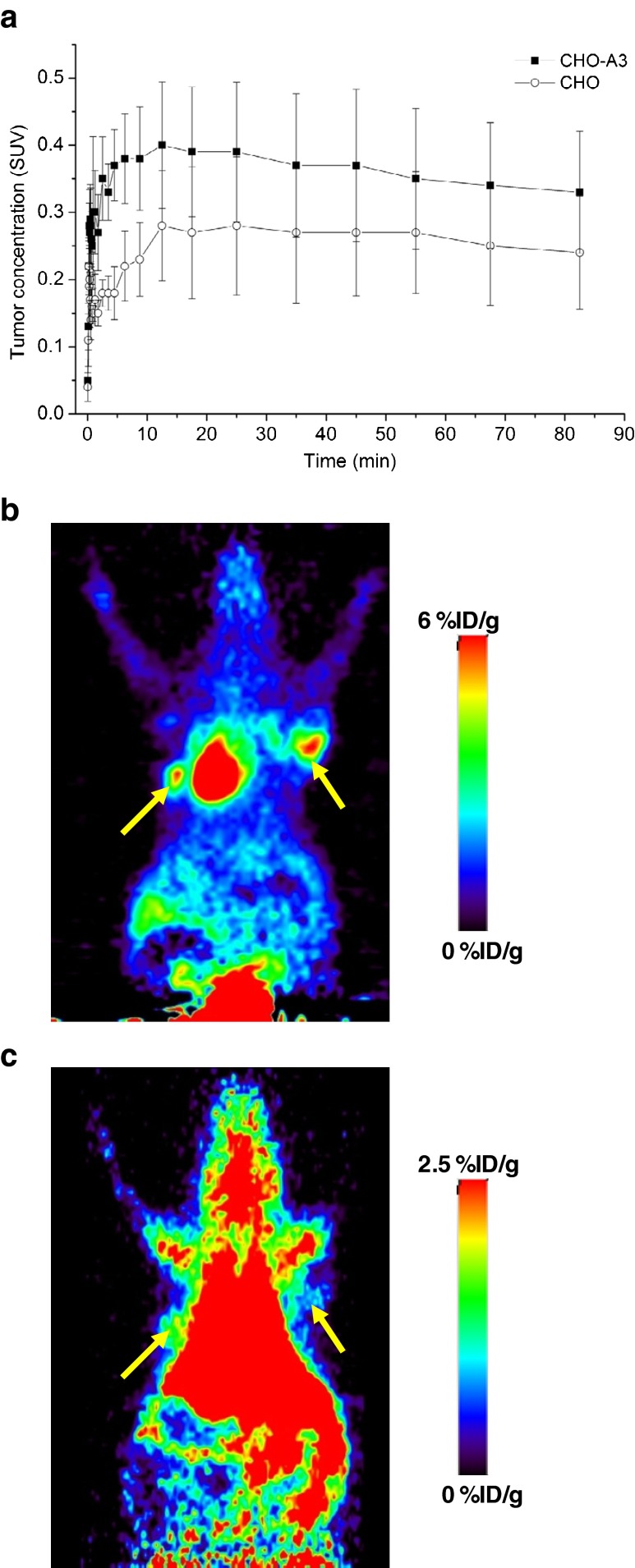
Uptake experiments in xenografted mice. **a** Time–activity curves (mean ± SEM) of [^18^F]FE@SUPPY in solid tissue masses resulting from bilateral cell inoculation of parental CHO cells and CHO-A3 cells. The solid masses from the injection of CHO-A3 cells show significantly higher tracer uptake than the solid masses from the injection of CHO cells (*p* = 0.03). **b** [^18^F]FDG PET summation (0 – 10 min) images obtained 1 h after tracer administration. **c** Typical [^18^F]FE@SUPPY PET summation (0–10 min) images. The respective radiation scales are indicated with the images

TACs of [^18^F]FE@SUPPY uptake in the solid tissue masses in mice injected with CHO or CHO-A3 cells are shown in Fig. 3a. The AUCs extracted from the TACs for mice injected with CHO-A3 cells showed an increased uptake of [^18^F]FE@SUPPY of around 1.42-fold (*p* = 0.03) in comparison with the AUCs for mice injected with CHO cells. [^18^F]FDG uptake in the mice injected with CHO-A3 cells was slightly higher than in those injected with CHO cells (6.3 %ID/g versus 5.4 %ID/g, respectively) but the difference did not reach statistical significance (*p* = 0.12). PET summation images after [^18^F]FDG administration and after [^18^F]FE@SUPPY administration are shown in Fig. 3b, c.

### Metabolism

#### Ex vivo stability

To assess the influence of pretreatment, radiometabolites of [^18^F]FE@SUPPY were determined by radio-HPLC in plasma and brain from rats pretreated with DMSO vehicle, MRS1523, FE@SUPPY or TQD. The amounts of radioactive metabolites were inversely proportional to the amounts of unchanged parent compound in both plasma and brain. At 60 min after tracer injection, plasma from rats pretreated with DMSO, TQD, FE@SUPPY and MRS1523 contained 25.8 ± 5.3 %, 39.9 ± 4.7 %, 32.4 ± 5.6 % and 26.5 ± 1.4 % of unchanged [^18^F]FE@SUPPY, respectively (Table 1). At 60 min after tracer injection, brain extracts from rats pretreated with DMSO, TQD, FE@SUPPY and MRS1523 contained 43.7 ± 9.5 %, 55.2 ± 7.0 %, 49.6 ± 9.4 % and 37.8 ± 4.3 % of unchanged [^18^F]FE@SUPPY, respectively (Table 1). However, there were no significant differences among the different groups, for both plasma and brain. Total recovery of radioactivity from blood and brain was 86 – 92 %.

**Table 1:** Metabolism of [^18^F]FE@SUPPY in blood and brain after pretreatment with DMSO, MRS1523, FE@SUPPY or TQD

Pretreatment	Unchanged [^18^F]FE@SUPPY at 60 min (%)		%ID/g at 60 min	
	Blood	Brain	Blood	Brain
DMSO	25.81 ± 5.3	43.73 ± 9.6	0.0216 ± 0.003	0.1132 ± 0.0002
MRS1523	26.54 ± 1.4	37.75 ± 4.3	0.0145 ± 0.002	0.0918 ± 0.0001
FE@SUPPLY	32.44 ± 5.7	49.64 ± 9.4	0.0160 ± 0.002	0.1273 ± 0.0000
TQD	39.87 ± 4.7	55.21 ± 7.0	0.0214 ± 0.004	0.1507 ± 0.0005

#### In vitro plasma stability

In human plasma, [^18^F]FE@SUPPY showed no degradation. In rat plasma, 60 min and 120 min after addition of [^18^F]FE@SUPPY, 18.7 ± 3 % and 35.0 ± 4 % of the radioactivity, respectively, was from metabolites The total recovery of radioactivity from the plasma samples was >95 %.

## Discussion

### General

A3R is involved in cardiovascular and neurological pathologies, and – as recently indicated – in tumour-related diseases. Since it has been reported that the A3R protein is expressed at particularly high levels in prostate, breast and colon carcinoma and melanoma, future clinical applications of A3R PET tracers can be envisaged, e.g. as imaging biomarkers for these disease with high mortality [1]. Surprisingly, no A3R antagonists have yet reached clinical trial status, although they are considered antiinflammatory, antiallergic and anticancer agents, and to have potential for the treatment of asthma, COPD, glaucoma and stroke [20]. Until recently, there were no PET tracers available for the imaging and noninvasive evaluation of A3R. Gessi et al. stated that “research in the A3 field is still in its infancy, with several important and challenging issues remaining to be addressed” [1]. Therefore, we developed [^18^F]FE@SUPPY as the first PET tracer for A3R. Radiopreparation and first preclinical evaluation including biodistribution, in vitro metabolic stability studies, ex vivo metabolism studies and in vitro autoradiography [2, 3, 6, 12, 13] showed quite promising results. This led us to the next step of the in vivo evaluation of [^18^F]FE@SUPPY under different conditions (baseline, specific blocking of A3R, blocking of P-gp, mice inoculated with human A3R cell xenografts) using small-animal PET and to assess in vitro tracer stability in human and rat plasma (an overview of the results of this evaluation of [^18^F]FE@SUPPY as a potential A3R PET tracer is presented in Table 2).

**Table 2:** Preclinical evaluation findings of [^18^F]FE@SUPPY and its potential for use in the clinical setting

	Findings	Final Countdown
Affinity for human A3R	4.2 nM [8]	Favourable
Radiosynthesis	Reliable, good specific activity [2, 3]	Favourable
Biodistribution in rats	Followed mainly A3 pattern (except testes) [3]	Good
Bone uptake in rats	None [3]	Favourable
Ex vivo metabolites in rats	Blood and brain	OK^a^
Stability in human and rat plasma	Stable over 120 min in human plasma	Favourable
Autoradiography in humans and rats	High selectivity; FE@SUPPLY comparable with MRS1523 [12]	Favourable
Xenograft model of human A3R	Specific uptake in CHO-A3 solid mass (*p* ≤ 0.03) compared to [^18^F]FDG	Favourable

^a^Metabolism in rats is six to eight times faster than in humans; therefore we assume that potential metabolites in humans would not be a problem, particularly since they are not radioactive [3]

### Small-animal PET

#### Baseline

The high degree of accumulation of [^18^F]FE@SUPPY in fat-rich regions might be explained by the high _HPLC_logD^7.4^ of 4.04 [6]. Surprisingly, baseline conditions in the brain found in this small-animal PET study did not reflect the increasing uptake of the tracer in brain, which we expected on the basis of a previous biodistribution study, in which we found that brain-to-blood ratios increase over time [3].

It is noteworthy that there were differences between baseline scans and vehicle scans: 1.35 times higher AUC for vehicle as compared to baseline without vehicle. The vehicle DMSO was necessary for dissolving the two A3R antagonists MRS1523 and FE@SUPPY for pretreatment (final injected volumes contained up to a maximum of 10 % DMSO). Tracer uptake in brain was increased by the presence of DMSO, which we explained by the fact that DMSO is a solubilizer, and therefore may enhance blood–brain barrier permeability.

#### Blocking

Blocking of [^18^F]FE@SUPPY with MRS1523 led to significant displacement of peak brain activity and AUCs (*p* = 0.009, *p* = 0.03), whereas FE@SUPPY pretreatment led to displacement that did not reach a significant level. This observation can be explained by the 5.31-fold higher affinity of MRS1523 for rat A3R (*K*_i_ 113 nM) compared with that of FE@SUPPY (*K*_i_ 600 nM) [21].

#### Efflux inhibition

TQD was administered at a dose that has been shown to completely inhibit P-gp at the blood–brain barrier [22], leading to 25 % increased brain uptake of [^18^F]FE@SUPPY in the brain relative to the vehicle treatment (*p* = 0.09). This suggests that P-gp efflux plays a role in limiting brain uptake of [^18^F]FE@SUPPY in rats.

#### Xenograft mouse model

[^18^F]FE@SUPPY showed significantly increased uptake in the tissue mass from CHO cells expressing the human A3R compared with the tissue mass from parental CHO cells (*p* = 0.03). This effect was not observed with [^18^F]FDG, although tissue masses from both transfected and parental CHO cells had high metabolic consumption due to their high proliferation rates. This indicates that, in contrast to [^18^F]FDG (*p* = 0.12), a gold standard tracer for the in vivo diagnosis of cancer and tumour diseases, [^18^F]FE@SUPPY is able to distinguish between parental CHO cells and CHO cells expressing the human A3R. The results in this preliminary xenograft model, keeping in mind the high affinity of [^18^F]FE@SUPPY for the human A3R (*K*_ih_ 4.2 nM), support the strategy of evaluating [^18^F]FE@SUPPY in “humanized models”.

### Metabolism

#### Ex vivo stability

The metabolic status (in vitro and ex vivo) of [^18^F]FE@SUPPY was characterized in a previous study [12]. For the in vivo experiments described here, an additional metabolic analysis was performed to exclude a possible influence of different blocking conditions on the uptake and metabolic behaviour of [^18^F]FE@SUPPY. Radioactive metabolites were found in both rat blood and rat brain under all four treatment conditions (vehicle, MRS1523, FE@SUPPY and TQD). However, all observed changes in metabolism were statistically insignificant, and a significant influence of the uptake caused by changes in metabolism can therefore be excluded. Nevertheless, plasma concentrations at 60 min after injection showed similar reductions to those after the blocking treatments in rat brains (36 % with MRS1523, 27 % with FE@SUPPY). The calculated uptake in plasma ranged from 0.014 ± 0.001 %ID/g (MRS1523) to 0.022 ± 0.003 %ID/g (vehicle); these values are not significantly different. Brain to blood uptake ratios ranged from 5.2 (baseline) to 7.9 (FE@SUPPY). Similar values were found in our biodistribution experiments (up to 5.4) [3].

Taking into account the data from the previous metabolic ex vivo study [12], in which we found 30 % parent compound left in plasma and no metabolites of [^18^F]FE@SUPPY in brain at 30 min after injection, and knowing that metabolism in rats is several times faster than in humans, we conclude that [^18^F]FE@SUPPY should be stable for the typical time-span of a clinical investigation in humans.

#### In vitro plasma stability

In rat plasma, degradation of [^18^F]FE@SUPPY led to 19 – 35 % radioactive metabolites over the observation period of 120 min, which is in a similar range to that observed ex vivo. In human plasma, [^18^F]FE@SUPPY was stable over the observation period of 120 min, which is encouraging for potential future applications in humans.

### Conclusion

It is known that A3R is available only at low densities in most tissues and at especially low densities in the central nervous system [23], and there are species differences regarding the A3R protein between rats and humans, resulting in reduced affinities of 1,4-dihydropyridine analogues (including MRS1523 and FE@SUPPY) for rat A3R. However, since A3R is involved in cardiovascular, neurological and tumour-related pathologies and could serve as an exceptional pharmaceutical target in the clinical setting, the purpose of the present study was the preclinical evaluation of [^18^F]FE@SUPPY in vivo.

[^18^F]FE@SUPPY showed high uptake in fat-rich regions and low uptake in brain due to a significant influence of efflux transporters. Therefore, we conclude that mechanisms involved in the observed tracer uptake in rat brain were partly specific (significantly blockable with MRS1523, blockable but not significantly with FE@SUPPY) and the rat animal model in general is not suitable for the evaluation of [^18^F]FE@SUPPY in vivo. Nevertheless, in xenografted mice [^18^F]FE@SUPPY showed significantly increased uptake in the tissue mass from CHO cells expressing human A3R compared with that in the tissue mass from parental CHO cells (*p* = 0.03). In contrast, [^18^F]FDG, the gold standard tracer for inflammation and tumour imaging, did not show significantly increased uptake in this model (*p* = 0.12) although tissue masses from both transfected and parental CHO cells showed similarly elevated levels of metabolic consumption due to their similar growth rate. This finding supports the strategy of evaluating [^18^F]FE@SUPPY in “humanized animal models”. Furthermore, no metabolites of [^18^F]FE@SUPPY were observed until 30 min after tracer administration in rat brain [12] and no metabolites were observed in human plasma in vitro. Since usually metabolism in rodents is several times faster than in humans, we expect [^18^F]FE@SUPPY to be stable for the typical time-span of a clinical investigation in humans. However, final proof of stability will only be provided in a human setting.

 In summary, as a final conclusion, the main findings of the preclinical evaluation of [^18^F]FE@SUPPY are shown in Table 2. All in all, they point in favour of the suitability and specificity of [^18^F]FE@SUPPY as a PET tracer for the noninvasive visualization of A3R and A3R-related pathologies in humans. Therefore, as a follow-up study we aim to evaluate [^18^F]FE@SUPPY in animal models with different implanted human tumour cell lines expressing A3R under baseline and blocking conditions.
